# Comprehensive Analysis of Alternative Splicing and Functionality in Neuronal Differentiation of P19 Cells

**DOI:** 10.1371/journal.pone.0016880

**Published:** 2011-02-18

**Authors:** Hitoshi Suzuki, Ken Osaki, Kaori Sano, A. H. M. Khurshid Alam, Yuichiro Nakamura, Yasuhito Ishigaki, Kozo Kawahara, Toshifumi Tsukahara

**Affiliations:** 1 Center for Nano Materials and Technology, Japan Advanced Institute of Science and Technology, Nomi, Ishikawa, Japan; 2 World Fusion Co., Ltd., Chuo-ku, Tokyo, Japan; 3 Graduate School of Materials Science, Japan Advanced Institute of Science and Technology, Nomi, Ishikawa, Japan; 4 Medical Research Institute, Kanazawa Medical University, Kahoku-gun, Ishikawa, Japan; Victor Chang Cardiac Research Institute (VCCRI), Australia

## Abstract

**Background:**

Alternative splicing, which produces multiple mRNAs from a single gene, occurs in most human genes and contributes to protein diversity. Many alternative isoforms are expressed in a spatio-temporal manner, and function in diverse processes, including in the neural system.

**Methodology/Principal Findings:**

The purpose of the present study was to comprehensively investigate neural-splicing using P19 cells. GeneChip Exon Array analysis was performed using total RNAs purified from cells during neuronal cell differentiation. To efficiently and readily extract the alternative exon candidates, 9 filtering conditions were prepared, yielding 262 candidate exons (236 genes). Semiquantitative RT-PCR results in 30 randomly selected candidates suggested that 87% of the candidates were differentially alternatively spliced in neuronal cells compared to undifferentiated cells. Gene ontology and pathway analyses suggested that many of the candidate genes were associated with neural events. Together with 66 genes whose functions in neural cells or organs were reported previously, 47 candidate genes were found to be linked to 189 events in the gene-level profile of neural differentiation. By text-mining for the alternative isoform, distinct functions of the isoforms of 9 candidate genes indicated by the result of Exon Array were confirmed.

**Conclusions/Significance:**

Alternative exons were successfully extracted. Results from the informatics analyses suggested that neural events were primarily governed by genes whose expression was increased and whose transcripts were differentially alternatively spliced in the neuronal cells. In addition to known functions in neural cells or organs, the uninvestigated alternative splicing events of 11 genes among 47 candidate genes suggested that cell cycle events are also potentially important. These genes may help researchers to differentiate the roles of alternative splicing in cell differentiation and cell proliferation.

## Introduction

The number of human genes (approximately 23,000 according to the Human Genome Project [1]) is just slightly higher than the number in lower eukaryotes such as worms. Most human exons are constitutive. However, >90% of human genes undergo in alternative splicing [2], a much higher percentage than that in lower eukaryotes [3]. Alternative splicing changes the use of exons, producing multiple types of transcripts from a single gene. Each transcript is translated into unique protein isoform. Alternative splicing enhances proteomic diversity to support complexity in higher eukaryotes [4]. Many alternative isoforms have distinct or opposing functions modified from another isoform, and play important biological roles [5], [6]. Alternative splicings often occur in a spatiotemporal manner, and some are regulated by alternative splicing regulators. In the case of neural tissues, several regulators, such as nPTB, Nova1, Fox-1, and their target pre-mRNAs have been identified [7]–[9]. Understanding alternative splicing networks is very important, because splicing is one of important gene expression processes and affected on gene expression network [10].

There are five basic types of alternative splicing that involve only the differential use of splice sites: exon skipping, 5′ or 3′ splice site (ss) selection, mutually exclusive, and retained intron [4]. In addition, 3′ss selection and/or the selection of poly-A additional signals help regulate alternative termination. Alternative promoters promote the differential use of the first exons adjacent to the transcriptional initiation sites, a process that is sometimes considered as an alternative splicing event [5], [11]. Alternative splicings can occur together, causing complicated splicing patterns. The differential use of exon, generated by alternative splicing events, including alternative termination and alternative promoters, generally changes some of amino acid codes of the expressed protein.

Several technologies, such as microarrays and high-throughput sequencing, have been developed to analyze alternative splicing in genome-wide scale [reviewed in [12]]. Junction and exon arrays are the two main microarrays to investigate alternative splicing employing probes specific to the exon(s). Exon array probes are designed for each exon, while probes of junction arrays are designed for exon-exon junctions. The GeneChip Exon Array produced by Affymetrix is a convenient assay in which operations from sample preparation to scanning are performed in a standard gene expression profiling environment [13]. Although various studies using Exon Array have been published, few have efficiently extracted alternative splicings from Exon Array results and validated by RT-PCR [14]–[19]. Even fewer reports have applied and analyzed the validated potential alternative exons using gene ontology (GO) or pathway analyses [18], [19].

P19 mouse embryonic carcinoma cells are a multi-potent cell type that can differentiate into either neural or cardio muscular cells depending on the retinoic acid (RA) concentration in the medium [20], [21]. We previously reported the regulation of neural-specific splicing of the Mef2c exon β in P19 cells [22]. The transcription factor Mef2 plays important roles in muscle differentiation and synaptogenesis [23], [24]. Fox-1 promoted the inclusion of Mef2c exon β via the GCAUG sequence [22]. In addition to Mef2c exon β and Fox-1, many alternative splicing events likely occur in a neural-specific manner during the neuronal differentiation of P19 cells. These splicing events are presumably regulated by splicing-regulators that are also expressed in a neural-specific manner. The alternatively spliced products, such as transcription factors, affect the transcriptional networks and function in neural events.

The goal of the present study was to comprehensively investigate neural-splicing using P19 cells. A GeneChip Exon Array was performed using total RNAs purified from undifferentiated P19 cells (Day 0), neuronal differentiated cells (Day 7), and cells from the early glial stage (Day 10). Nine filtering conditions were used for probe sets based on their annotations, estimated gene expression levels, splicing index (SI), detection above back ground (DABG) values, and predictions of alternative splicing. A total of 262 differentially alternatively spliced (DAS) candidate exons of neural splicing were extracted. Thirty exons among the 262 DAS exons were validated by RT-PCR. The RT-PCR experiments suggested that 87% of these exons were actually changed between undifferentiated and neuronal differentiated cells. This result indicated the method of the candidate exons extraction was very successful. Results from informatics approaches, including GO analysis, text-mining, and pathway analysis, also suggested that many of the candidate exons were related to neural events. In addition to gene-level informatics analyses, text-mining for the alternative isoform were performed. This informatics analysis is a new trial to identify exon-level annotations.

## Methods

### Cell culture and RNA purification

P19 cells were maintained in Minimum Essential Medium (MEM) α Medium (α-MEM; Sigma) supplemented with 10% fetal bovine serum (FBS; Sigma) [25]. To induce neuronal cell differentiation, P19 cells (1×10^5^ cells/mL) were treated with 1 µM all trans-retinoic acid (RA) for 4 d in a 10-cm petri dish (Falcon) with α-MEM containing 10% FBS as described previously [22], [25]. Total RNAs were collected from undifferentiated cells (Day 0), cells during neuronal differentiation (Day 1, 4, 7), and from cells in the early glial stage (Day 10) using RNeasy (Qiagen).

### Exon array

Both cDNA and cRNA syntheses were performed as described in the supplier's instructions. Total RNAs from undifferentiated (Day 0), neuronally differentiated P19 (Day 7), and early glial stage cells (Day 10) were applied to a GeneChip Mouse Exon 1.0 ST Array (Affimetrix). Hybridization, washing and scanning were performed on a fluidics station and a GeneChip scanner (Affimetrix) according to the supplier's instructions. The accession number of the Exon Array data is GSE23710.

### Estimation of signals

Signal data of the CEL files were sketched, normalized, and summarized with the PLIER method for exon-level intensities and with the Iter-PLIER for gene-level intensities using the Affymetrix Expression Console. The “core” option was used in both cases, which limits analysis to RefSeq-mapped probesets and to the mRNA of full-length CDS. The presence or absence of each probe was determined by the DABG *p*-values. Signal estimates of 3 time points (Day 0, 7, and 10) with biological duplicates were summarized with various annotations supplied by Affymetrix, such as probeset positions, transcript accessions, and probe sequences.

### Extraction of the candidate exons and genes

Although multiple probesets are designed within the same exon, probeset intensities represent the expression levels of exons. The ratio of the probeset intensity to the gene intensity, designated as the normalized intensity (NI), was estimated in 3 time points (NI_d0_, NI_d7_, and NI_d10_). The splicing index (SI) was calculated by taking the log ratio (base 2) of the averaged NIs of biological duplicates [19], [26]. Probesets annotated as having a high potential of cross-hybridization were removed from the analysis. The
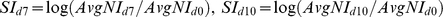
splicing patterns of exons were predicted from using the UCSC Blast-like alignment (blat) search tool (http://genome.ucsc.edu/cgi-bin/hgBlat). An automatic system of alternative splicing prediction was created using the UCSC blat and NCBI BLAST (Basic Local Alignment Tool) searches. A detailed explanation of the candidate exon extraction process is described in the Results section (see also Table 1).

**Table 1:** Procedures applied to extract alternative exon candidates.

		Exons*	Genes
Filtering Conditions		# of Probesets and Gene Probesets	
	Estimated signals using CoreGene and CoreExon by Expression Console	221,336	16,661
1.	Remove probesets that have Max. or Min. number of ‘probeset ID start’ in each gene	188,374	15,753
2.	Remove probesets that indicate possible UTRs (Length of probeset ID sequence <500 nt)	185,028	15,654
3.	Remove probesets that cause non-specific adsorption (x-hybr; FALSE)	159,552	15,238
4.	Keep the genes expressed on both Day 0 and Day 7	102,661	8,601
5.	Keep the exons expressed on either Day 0 or Day 7 or both (*p*≤0.05 in DABG)	94,780§	8,541
6.	Keep probesets that significantly change their expression between Day 0 and Day 7 (ABS_SI_d7_ ≥1.35)	1,636	948
7.	Remove a probeset if both of its neighbors change expression	1,107	805
8.	Keep probesets that indicate possible alternative exons in database (ucsc blat search)	309	278
9.	Keep a probeset that its ABS_SI_d7_ value is larger than its ABS_SI_d10_ value	262	236

* The probeset likely indicate an exon, although multiple probesets may exist in an exon.

**Figure 1:**
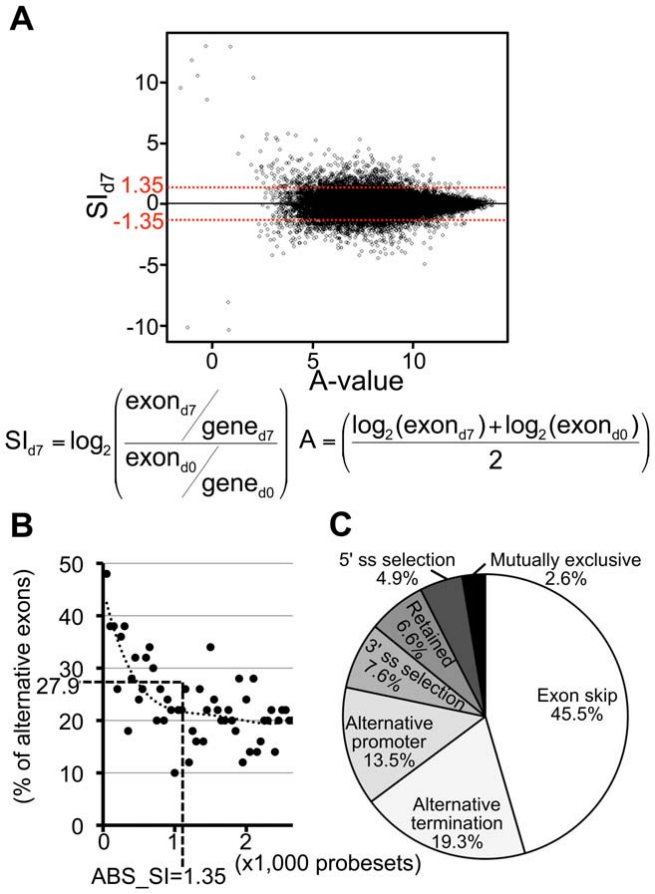
Statistical analyses of the extraction of candidate exons. (A) SI-A plot for 94,780 probesets after the fifth procedure in Table 1. Vertical and horizontal axes show SI_d7_ values and A-values, respectively. (B) Percentage of the predicted alternative exon. The 2,650 probeset ID sequences whose ABS_SI_d7_ values were >1.0 (see also sixth procedure of Table 1) were assessed for whether they indicated the alternative exon. Dots indicate percentages of the alternative exon in each group of 50 probesets from the highest ABS_SI_d7_ to approximately 1.0. Dotted line indicates the approximation curve. Vertical broken line indicates the threshold (ABS_SI_d7_ = 1.35) in Table 1. Horizontal broken line (27.9%) indicates the percentage of the predicted alternative exon. (C) Percentage of alternative splicing types in the DAS exons. The types of alternative splicings of 262 probeset ID sequences were searched using UCSC blat search.

§ Signals of these probesets were plotted using the SI_d7_ value (Figure 1). Note: In the fourth procedure, genes were retained if ≥50% of probesets in each gene had *p*≤0.05 in DABG. In the seventh procedure, according to the of probeset ID start position, upstream and downstream neighbor probesets were searched whose expression was detectable (DABG; *p*≤0.05). If ABS_ SI_d7_ value of the neighboring probeset was ≥0.667, the neighbor was considered to have changed expression. A probeset was removed if both of its neighbors changed expression. If a probeset with only one neighbor, the probeset was removed if that neighbor changed expression. In the ninth procedure, in addition to comparing of the ABS_SI values, a probeset was retained when its SI_d7_ and SI_d10_ were a negative number and ≥1.35, respectively, or when its SI_d7_ and SI_d10_ are a positive number and ≤−1.35, respectively.

A gene was defined as “expressed” when ≥50% of probesets of the same gene had a DABG *p*-value of ≤0.05 in both biological duplicate of the same time point [19], [26]. Genes that were defined as “expressed” on both Day 0 and 7 or on either Day 0 or 7 were analyzed. For gene-level expression analysis, these “expressed” genes were categorized as differentially expressed upregulated (DEX-up) or downregulated (DEX-down). A gene was categorized in the DEX-up group if its expression level increased ≥2-fold and ≤10-fold from Day 0 to Day 7, and decreased from Day 7 to Day 10. A gene was categorized in the DEX-down group if its expression decreased ≥2-fold and ≤10-fold from Day 0 to Day 7, and increased from Day 7 to Day 10.

### Semiquantitative RT-PCR

The total RNAs of P19 cells were prepared as described above. Total RNAs from mouse tissues were obtained from a commercial source (Ambion). The cDNAs were made from 2 µg of total RNAs using SuperScript III (Invitrogen) and 0.5 µg of oligo dT primer in 20-µL reaction mixture. The cDNAs are used as templates for the PCR. The PCRs were performed using the GoTaq polymerase (Promega) with specific primers. The DNA primer sequences, number of cycles, and annealing temperatures for the candidates are described in Table S1. Those for *β-Actin* and *GluR1* were described in a previous report [22]. The PCR products were analyzed on a 6% polyacrylamide gel. The gels were stained with SYBR Green I (Takara), and the images were analyzed in LAS-3000 (Fuji Film). The sequences of the PCR products are confirmed in the 3100 DNA sequencer (ABI). Accession numbers of sequences for novel alternative variants are shown in Table 2. Densitometry was performed using MultiGauge v 3.0 software (Fuji Film). Each experiment was performed ≥3 times to confirm reproducibility.

**Table 2:** Validation of alternative splicings in neuronal differentiation as detected by RT-PCR.

Gene	Probeset ID	SI_d7_	Exon type	Neural AS	Change of PCR product (%/%)	Length (nt)	Novel splicing
*Abi2*	4987109	1.67	Exon skip	**+**	7.44 Hold d7/d0 of 329nt	329, 146	-
*Aff3*	5291502	3.81	Exon skip	**++**	53.87 Hold d7/d0 of 178nt	178, 102	-
*Ank3*	5060679	−3.30	3′sss	**+**	4.78 Hold d0/d7 of 703nt	703, 115	-
*Aplp2*	5477899	2.04	Exon skip	**+**	5.88 Hold d7/d0 of 197nt	197, 157	-
*Arhgef12*	5209805	−1.44	Alt terminater	**-**	1.90 Hold d0/d7 of 111nt	166, 111	-
*Atf2*	5194737	1.85	Exon skip	**+**	5.45 Hold d7/d0 of 335nt	335, 272, 195, 132	AB575970
*Atp1a3*	5385869	−2.71	Retained intron	**++**	16.88 Hold d0/d7 of 446nt	446, 109	-
*Bai2*	5286600	−2.05	Exon skip, Mutual	**+**	5.20 Hold d7/d0 of 173nt	209, 173, 110	-
*Bcl11a*	4969426	−1.43	5′sss, Alt terminater	**+**	2.82 Hold d7/d0 of 153nt	201, 153, 101	-
*Clta*	4399984	3.05	Exon skip, Mutual	**+**	5.93 Hold d7/d0 of 204nt	204, 168, 150, 114	AB575971
*Dlg3*	5344442	5.39	Exon skip, Mutual	**++**	52.04 Hold d7/d0 of 198nt	252, 198, 156, 152	AB575972
*Epb4.1l3*	5517310	2.76	Exon skip	**+**	5.78 Hold d7/d0 of 264nt	339, 264, 141	-
*Epb4.1l3*	4866829	2.04	Exon skip	**+**	2.54 Hold d7/d0 of 224nt	224, 127	-
*Fam113a*	4648309	−2.98	Exon skip, 3′sss	**+**	3.74 Hold d7/d0 of 102nt	363, 182, 102	AB575973
*Fchsd2*	4408143	3.36	Exon skip	**++**	19.52 Hold d7/d0 of 235nt	235, 163	-
*Gnao1*	4489479	−1.62	3′sss, Alt terminater	**+**	2.12 Hold d0/d7 of 189nt	189, 124	-
*Kif1b*	4352234	2.15	Exon skip	**+**	5.70 Hold d7/d0 of 193nt	235, 193, 157, 115	AB575974
*Kif21b*	5130589	2.85	Exon skip	**++**	111.60 Hold d7/d0 of 311nt	311, 131	-
*Kif2a*	5345537	4.23	Exon skip	**++**	135.44 Hold d7/d0 of 231nt	231, 117	-
*Neo1*	5491604	1.77	3′sss	**+**	6.84 Hold d7/d0 of 153nt	153, 105	-
*Neo1*	4604088	5.90	Exon skip	**++**	31.00 Hold d7/d0 of 151nt	151, 118	-
*Plekha5*	5343636	1.59	Exon skip, 5′sss, 3′sss	**++**	17.93 Hold d7/d0 of 346nt	346, 230, 154	AB575975
	4740762	2.06					
*Rbm9*	5479607	2.04	Alt promoter	**+**	7.41 Hold d0/d7 of 251nt	251, 157, 134	-
*Rnf138*	4323558	2.31	Exon skip	**-**	1.17 Hold d7/d0 of 317nt	317, 209	-
*Rufy3*	4321833	−1.85	Exon skip, Alt promoter	**+**	2.01 Hold d0/d7 of 157nt	157, 103	-
*Snap91*	4960676	2.78	Exon skip	**++**	14.98 Hold d0/d7 of 162nt	246, 162	-
*Tmem219*	5381697	−1.91	Alt promoter, Exon skip, 3′sss	**-**	1.58 Hold d0/d7 of 293nt	263, 94	-
*Tpd52l2*	4473030	1.86	Exon skip	**++**	12.47 Hold d7/d0 of 185nt	185, 158, 116	-
*Tprkb*	5267179	−2.75	Exon skip	**-**	1.00 Hold d7/d0 of 104nt	188, 104	-
*Zfp326*	5023736	2.31	Exon skip, 5′sss, 3′sss,	**+**	2.94 Hold d0/d7 of 123nt	529, 426, 265,	AB575976-
			Retained intron			240, 123	AB575978

Percentages of the relative amounts of alternative exons were compared between Day 7 (D7) and Day 0 (D0). (−) Changes is <2-fold, or the change on Day 10 is larger than Day 7, (+) Change is >2-fold but <10-fold, and (++)Change is >10-fold. sss, Splice site selection.

### Gene ontology analysis, pathway analysis and text-mining

The GO analyses were performed for genes with candidate exons and for genes in the DEX-up and DEX-down groups [27]. Statistically-significant biological process terms were obtained with Fisher's Exact Test (*p*≤0.01) using PathwayStudio® (Ariadne Genomics, Inc, Rockville, MD, USA) [27], [28].

## Results

### Extraction of the alternative exon candidates

Because probes of “core” exons was used, most of the analyzed probes were constitutive exons. Nine filtering conditions were constructed to extract the candidate exons of alternative splicings (Table 1). The filtering procedures (including our original procedures) were arranged by considering the general features of alternative splicing and previous concepts of exon extraction [26]. Because many outermost probesets are designed at potential exons outside of the genes, the first and last probesets of each gene were removed. Since the length of most human exons is <300 nucleotides (nt) [29], probesets with sequence of >500 nt were excluded. The long probesets mostly indicate untranslated region (UTR). Probesets with an annotation of a high potential for cross-hybridization were also removed. After filtering with these conditions, 221,336 probesets of core exons of 16,661 genes were reduced to 159,552 probesets of 15,238 genes (Table 1, third procedure).

To identify alternative splicings, genes should be expressed on both Day 0 and Day 7. The reliability of gene expression level was determined by the DABG *p*-values of the probesets (see Methods) [19], [26]. Probesets of genes expressed on both Day 0 and Day 7 were retained on the list (Table 1, fourth procedure). In addition, exon expression level should be detected at Day 0 or Day 7. Probesets with in a DABG *p*-values of ≤0.05 on Day 0 or 7 were accepted for the next filtering procedure. These filtering conditions reduced the number of probesets to 94,780 (Table 1, fifth procedure).

The splicing index SI of a probeset represents its change in exon expression, which is adjusted according to its gene-level expression. The A-value is the average intensity between Day 0 and 7. SI_d7_ values and A-values of 94,780 probesets are plotted in Figure 1A. Most probesets were closely plotted in the middle (SI_d7_ = 0.00). This result indicated that probesets with a small absolute value of SI_d7_ (ABS_SI_d7_) were not related to the alternations in exon usage between Day 0 and 7. A threshold ±1.35 of SI_d7_ (2.55-fold) was set, and probesets with ≥1.35 ABS_SI_d7_ were retained on the list (Table 1, sixth procedure).

SI of probeset represents a change of exon expression, which is adjusted with its entire gene level expression. However, Because the SI of a probeset cannot indicate the relationship between adjacent exons, the difference in SI_d7_ values was assessed in a local area of the transcript. We separately extracted the adjacent downstream or upstream probesets that were surely expressed (no cross-hybridization, DABG *p*-value ≤0.05). When ≥1 of the adjacent probesets had an ABS_SI_d7_ value of ≤0.667, the probesets were retained on the list (Table 1, seventh procedure). Theoretically the probeset indicates a border between alternative and constitutive exons after this filtering condition. If a probeset were kept only when both adjacent probesets had an ABS_SI_d7_ value of ≤0.667, then complex splicing events would be accidentally removed.

Since most the probesets were designed to constitutive exons, it is possible that many constitutive exons are involved in the 1,107 exons that were left after the seventh filtering (Table 1). We searched these probesets' sequences in the UCSC blat and found alternative exons. The probeset was categorized as a candidate alternative exon. When a probeset sequence resulted in multiple hits for mRNA fragments in Genbank and EST and also skipped other multiple fragments in the range of each gene on genomic structure, then the probeset was categorized as an alternative exon. An automatic system was used to a double check these sequences. Contradictory exons were manually curated in the UCSC blat search again. Approximately 28% of the probesets (309 probesets) were predicted to be alternative exons (Figure 1B & Table 1, eighth procedure).

Finally, the SI_d7_ and SI_d10_ values were compared. A probeset was retained if its SI_d7_ was larger than SI_d10_ when SI_d7_ was positive (or smaller than its SI_d10_ value when SI_d7_ was negative) (Table 1, ninth procedure). Altogether, 262 probesets containing all alternative splicing types were extracted (Table S2 & Figure 1C). These probesets were identified as DAS exons that potentially had undergone alternative splicing in a neural-specific manner.

### Semiquantitative RT-PCR for the candidate exons

**Figure 2:**
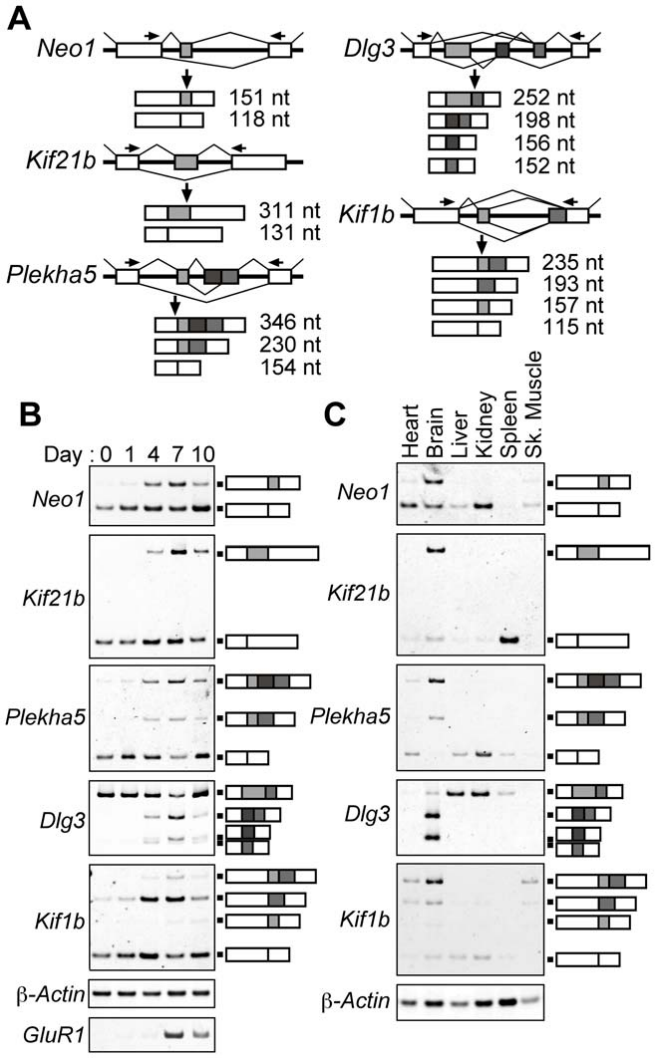
Semiquantitative RT-PCR experiments. (A) Schematic representations of alternative splicing. Thirty exons were randomly selected from 262 DAS exons. Five of 30 exons (*Neo1* exon17, *Kif21b*, *Plekha5*, *Dlg3*, and *Kif1b*) are shown; the other exons are shown in Figure S1. Boxes and middle lines indicate exons and introns, respectively. Gray indicates possible alternative exon. Arrows indicate the locations of primer annealing sites. Numbers indicate the length of PCR products. The sequences of PCR products were confirmed by sequencing analysis. Semiquantitative RT-PCR in P19 cells (Day 0, 1, 4, 7, and 10) (B) and in adult mouse brain and other tissues (C). *β-Actin* was used as a control. *GluR1* was used as a neural differentiation marker (B only). Schematic representations of the PCR products are shown on the right side of the panels.

To examine whether the potential DAS exons are actually involved in alternative splicing during the neuronal differentiation of P19 cells, semiquantitative RT-PCR was performed. Semiquantitative RT-PCR is more appropriate than qPCR in the analysis of alternatively spliced products. Among 262 DAS exons, 30 exons were randomly selected (Figure 2A & Figure S1A). Typical patterns for the DAS events in P19 cells are shown in Figure 2B. Densitometric analysis showed alternative splicing of 10 exons changed >10-fold between Day 0 (undifferentiated P19 cells) and Day 7 (differentiated P19 cells). The changes in the alternative splicing of the other 16 exons were >2-fold but <10-fold (Table 2 & Figure S2B). The expressions of these 26 exons increased or decreased consistent with the directions indicated by SI_ d7_. The changes of these 26 exons on Day 7 were greater than those on Day 10, consistent with the results in Table 1 (ninth procedure). These results show that neuronal cell stage-specific alternative splicing occurred in these 26 of 30 exons, and suggested that 87% of the 262 DAS exons were involved in neural splicing.

Seven alternative splicing events had novel splicing patterns (*Kif1b*, *Dlg3*, *Plekha5*, *Atf2*, *Clta*, *Fam113a*, and *Zfp326*; Table 2) in mouse. The patterns included a novel exon, novel splice sites, and novel combinations. Except for the novel *Dlg3* variant, the other 6 variants were detected in adult mouse brain (Figure S2C). Among 26 alternative exons, the percentages of alternative variants of 17 exons were changed >2-fold in the brain compared to other tissues, which showed expression patterns similar to those in the brain (Figure 2C & Figure S1C). Although the differentiation of P19 cells is a model system, this result implied that alternative splicings of more than half of DAS exons had similar regulations between neuronal differentiated P19 cells and brain.

### Gene ontology analysis of the candidate exons

**Figure 3:**
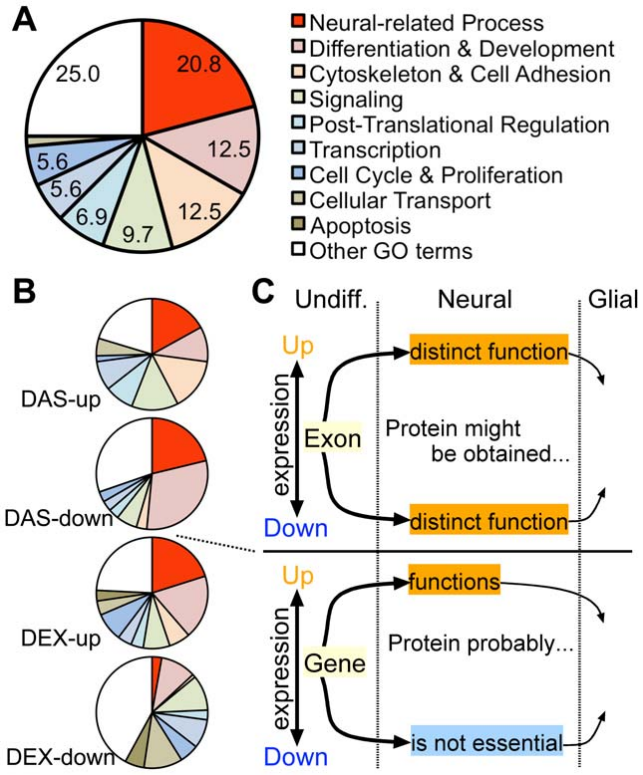
Gene Ontology analysis. (A) GO analysis in 262 DAS exons (236 DAS genes). Seventy-two GO terms (*p*-value ≤0.01; Fisher's *t*-test) were found from the 236 DAS genes. These terms were categorized into 10 groups. The percentage of the GO terms is shown. (B) The GO analysis at the DAS and DEX genes. The 236 DAS genes were divided into DAS-up (153 genes) and DAS-down (88 genes) groups, according to whether the exon expression levels were elevated or reduced, respectively, on Day 7 compared to Day 0. DAS-up and DAS-down groups were examined in GO analysis. The gene expressions of 1,666 DEX-up genes were elevated (from 2- to 10-fold) on Day 7 compared with Day 0, and decreased from Day 7 to Day 10. The gene expressions of 550 DEX-down genes were reduced (from 2- to 10-hold) on Day 7 compared with Day 0, and increased from Day 7 to Day 10. DEX-up and DEX-down genes were separately analyzed in GO analysis. (C) Primary relationship between expression profile and GO terms. The DEX-down genes showed markedly fewer neural-related GO terms. The DEX-up and DAS genes are primarily responsible for the neural-related GO terms, and seem to be equivalent to other events occurring in the neuronal differentiation in P19 cells.

To test whether the 262 DAS exons of 236 DAS genes were involved in neural events, GO analysis was performed using the Biological Process category. A total of 72 GO terms (*p*≤0.01, Fisher's Exact test) were obtained from the 236 DAS genes. These terms were classified into 10 groups: neural-related process (there were no neural-related terms in other terms), differentiation and development, cytoskeleton and cell adhesion, signaling, post-translational regulation, transcription, cell cycle and proliferation, cellular transport, apoptosis, and others. The neural-related process group was the most abundant, accounted for 20.8% of all terms, followed by differentiation and development (12.5%), cytoskeleton and cell adhesion (12.5%), and signaling (9.7%) (Figure 3A).

The 262 DAS exons were categorized into two groups according to whether the SI_d7_ value was ≥+1.35 (DAS-up; 153 genes) or ≤−1.35 (DAS-down; 88 genes). The percentage of neural-related GO terms in the DAS-up and DAS-down groups were 17.0% and 21.2%, respectively (Figure 3B). This result suggests that the protein isoforms translated from these alternatively spliced transcripts, which were either included or excluded exons, were involved in neural events.

Affymetrix Expression Console calculates the expression level of each gene based on the intensities of probesets of the Exon Array. We analyzed the gene expression of the same samples and obtained the differentially expressed (DEX) genes in P19 neuronal cells. The expression of 1666 DEX-up genes increased ≥2-fold and ≤10-fold from Day 0 to Day 7, and decreased from Day 7 to Day 10 (Table S3). The DEX-up gene group had 194 GO terms, including neural-related process (20.1%) and differentiation and development terms (18.6%) (Figure 3B). The DEX-up and DAS gene results were quite similar, suggesting that genes in both groups were related with neural-events (Figure 3C).

A total of 550 DEX-down genes were obtained, whose expression levels decreased ≥2-fold and ≤10-fold from Day 0 to Day 7 and increased from Day 7 to Day 10 (Table S4). The DEX-down genes had 107 cell process GO terms. The percentage of neural-related processes in all 107 terms was only 2.8%, and those of cellular transport and apoptosis were 11.0% and 5.6% (Figure 3B). The GO terms of DEX-down genes showed unique patterns compared with those of the DAS, DAS-up, DAS-down, or DEX-up genes, suggesting that the DEX-down genes are not responsible for neural-events (Figure 3C).

### Potentially important exons extracted by pathway analysis and text-mining

**Figure 4:**
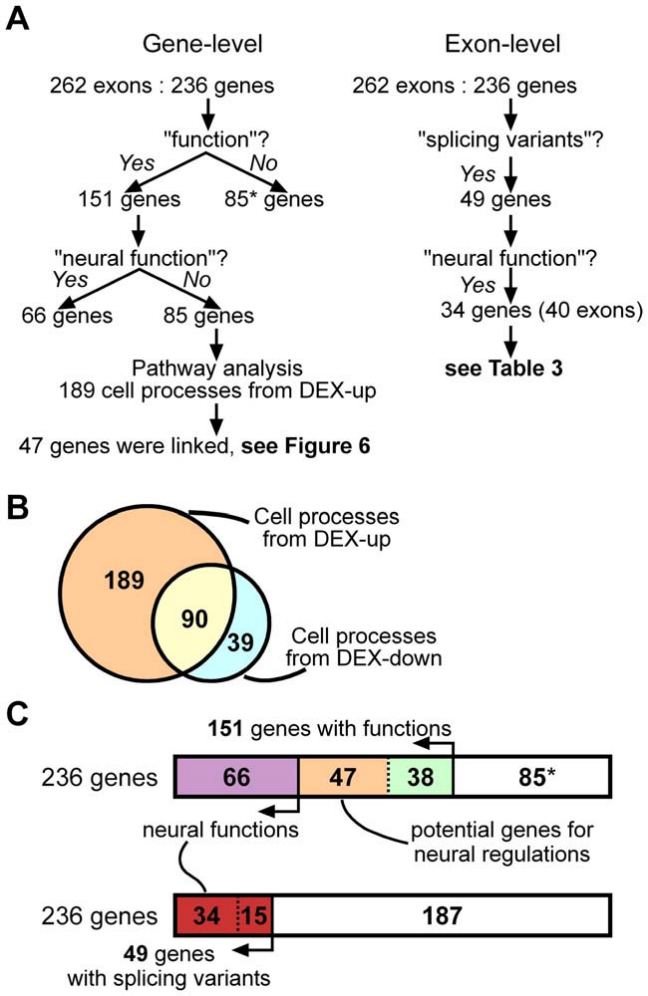
Pathway analysis and text-mining. (A) A Flow chart of informatics analyses at the gene-level (left) and exon-level (right). PathwayStudio® was used on 236 DAS genes. The functions of 85 genes were not reported in multiple studies, and these were categorized as unknown genes (*). Sixty-six genes were reported in studies on the functions in neural cells or organs, and were categorized as well-known genes. Another 85 genes were categorized as functional genes. The approach for the 85 functional genes is described below. Among the 236 DAS genes, 49 were reported in articles on ‘splicing variant’ or ‘isoform’. Thirty-four genes overlapped with the 66 well-known genes. Further confirmation was performed (Table 3). (B) Number of cell processes in pathway analysis of DEX-up and/or DEX-down genes (see Figure 3C). Orange, blue, and yellow indicate the number of biological processes from DEX-up genes, DEX-down genes, or both, respectively. Since DEX-up genes are primarily important for neural-events and DEX-down genes are not (see Figure 3), it is predicted that 189 biological processes take place in the neural differentiation. Forty-seven of the 85 functional DAS genes from the remaining 170 genes, whose functions matched with the 189 specific biological processes of the DEX-up group, were extracted. (C) Number of genes in Figure 4A. Upper box shows 66 well-known (purple), 85 unknown (*), and 85 functional genes. Among the 85 functional genes, 38 genes were not linked (green) and 47 genes were linked with the 189 processes and were categorized as potential genes for neural regulation (orange). Among the 236 DAS genes, 49 genes (red) were found in articles concerning ‘splicing variant’ or ‘isoform’ (lower box) and 34 genes were found in articles of neural functions.

Text-mining and pathway analysis were performed for the DAS genes using MedScan® text-mining technology in PathwayStudio® (Ariadne Genomics) [28]. Of the 236 DAS genes, the functions (cell process) 151 genes have been reported in multiple publications. The remaining 85 genes were categorized as ‘unknown genes’. Of the151 DAS genes with known functions, 66 were categorized as ‘well-known genes’ in neural processes since their functions in neural cells or organs were reported previously. The remaining 85 were categorized as ‘functional DAS genes’, having little reported evidence of neural regulatory roles (Figure 4A & 4C). This suggests that many DAS genes are related with neural events, as already shown by GO analyses. Because 66 well-known genes has been studied in neural cells or organs, further research about functional differences between alternative isoforms will probably clarify their functions in non-neural cells or organs. It will be very difficult to analyze alternative isoforms in the 85 unknown genes, since there are too few clues about their functions. Therefore, we focused on the 85 functional DAS genes.

In order to extract potentially important genes from the 85 functional DAS genes, we focused on DEX-up genes since DEX-up genes and DAS genes might affect on their expressions each other. As shown in GO analysis (Figure 3), in contrast to the DEX-down genes, many of the DEX-up genes seemed to regulate neural events. A total of 279 cell process terms closely related to DEX-up genes were extracted from the literature using text-mining and PathwayStudio® database (Figure 4B). Ninety overlapping terms of DEX-up and DEX-down groups were subtracted from the 279 terms. The remaining 189 cell process terms are presumed to be responsible for events in the neuronal P19 cells.

**Figure 5:**
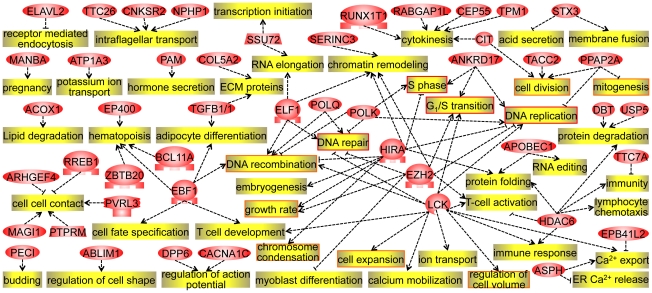
Pathway analysis of 47 DAS genes. PathwayStudio® was used to find linkages between the 85 functional genes and 189 biological processes (see Figure 4A). Forty-seven DAS genes and their linked to biological processes are shown. Cell cycle–related functions were found (orange & red), especially those relate to the G_1_ and/or S phase (red).

We next extracted biological relationships between the 189 cell processes and 85 functional DAS genes. Forty-seven DAS genes were linked to these cell processes (Figure 4A & Table S5). These 47 genes are potentially important, because isoforms created by neural splicing may acquire novel functions that have not been reported in neural cells. Eleven genes out of the 47 genes were involved in cell cycle-related events, processes in the G1 and/or S phase (Figure 5). This result is reasonable, since cell proliferation and cell differentiation are very closely related.

### Informatics and experimental analyses of splicing isoforms

In addition to the above gene-level analyses, we tried to perform exon-level informatics analyses. Among 236 DAS genes, 49 genes have been reported in articles about isoforms or splicing variants of previously investigated transcripts (Figure 4C & Table S2). Thirty-four genes were overlapped between the 66 well-known genes and 49 genes with known splicing (Figure 4A & 4C). The exons of 15 of the 34 genes were consistent with previous reported variants (Table 3). Of the 15 genes, distinct functions of the alternative isoforms in each gene were confirmed in 9 genes, including *Gnao1* (Table 3). These might mediate neural events as a result of alternative splicing. For instance, results of semiquantitative RT-PCR and the SI value of *Gnao1* showed that the *Gnao1* B isoform (189 nt) changed to the A isoform (124 nt) in P19 neuronal cells (Table 2 & 3). This result is consistent with a previous report showing that GNAO1 A isoform, but not the B isoform, is required for the light response of retinal bipolar cells [30].

**Table 3:** Exon-level annotations of 34 genes with known splicing.

	“Exon”	Reported regulations	Position indicated by	Isoform name		Distinct function of the isoform	
Protein	Probeset ID	in neural cells or organs	“Probeset”-“Article”	with/without “Exon”	SI_d7_	Function	PMID
DTNA	5566371	regulation of synapse, etc	same	α-dystrobrevin-2/1	1.56	viral life cycle	12475945
EPB4.1	5594543	cytoskeleton assembly, etc	same	Epb 4.1 variant1/2	2.70	cytoskeleton assembly	9092575
	5012146		same	Epb 4.1 variant1/3	1.50		9092575
GNAO1	4489479	neurotransmitter uptake	same	Gnao1B/A	−1.62	neurotransmitter uptake	12077185
GPHN	4505349	synaptic transmission, etc	same	Gphn2,4,6/2,6	−1.74	Mo-cofactor synthesis	18411266
KITL	5091217	neurogenesis, etc	same	SCF248/220	1.47	proteolysis	7507105
MTAP2	5123064	cytoskeleton assembly	same	Map2s/c	13.22	neuclear localization	10383434
	5161361	neurite outgrowth, etc.	same	HMW/LMW map2	−3.24		10383434
NFASC	5439373	cell contact,	same	NF186/155	2.63	neurite outgrowth	16314110
	4436418	cell adhesion	same		2.11		16314110
PITX2	4556486	morphogenesis	same	Pitx2c/a	−1.42	morphogenesis	10662647
THRA	4582355	Glucose metabolism,	same	c-erbA α1/2	−1.79	DNA binding, etc	2901090
	4705058	neurogenesis, etc.	same	c-erbA α2/3 (&1)	1.44		2901090
ABCC5	5507375	relaxation	same	ABCC5 SV1, 2, 3/	2.62	not found	N/A
CSF1	5305033	microglia activation, etc	same	Csf1 variant3/1	−2.75		
CUGBP2	4579194	RNA splicing, etc	same	Napor-3/Etr-3	−1.59		
NRCAM	5034147	axon guidance, etc.	same	Nrcam variant1/2	11.80		
SYT7	5121401	vesicle exocytosis,	same	Syt7 variant3 (g)/1	2.58		
	4667795	endocytosis, etc	same	Syt7 variant2/1	2.60		
EPB4.1L3	5517310	organogenesis	same	not determined	2.76		
	4866829		same		2.04		
APLP2	5477899	axon cargo transport, etc	different	Aplp2 variant2/1	2.04	chondloitin modification	17371289
GAS7	4941178	cell growth, etc.	different	Gas7a/b	2.50	neurite outgrowth	15948147
RBM9	5479607	RNA splicing	different	Fox-2f/a	2.04	RNA splicing	17715393
TCF4	4886763	neuron differentiation	different	Mitf-2a/b	1.85	transcription	8631961
CACNA1B	4683815	synaptic transmission, etc	different	Cacna1b variant1/2	−1.35	not found	N/A
CLTA	4399984	neurotransmitter secretion	different	LCa variant2 (&3)/1	3.05		
DLG3	5344442	synaptogenesis	different	Dlg3 variant1/2	5.39		
TSC2	5151584	S phase, etc.	different	Tsc2 isoform 5/4	1.87		
EGFR	5316652	axonogenesis, etc.	different	Egfr variant2 (&4)/1	2.96		
BCL2L11	5411420	neuronal death, etc	different	undetermined	2.26	N/A	
ENPP3	4509455	cell differentiation	different		3.16		
	4522439		different		2.38		
FRAP1	4375053	synaptic plasticity, etc	different		1.78		
MBNL1	4357388	reflex	different		−2.16		
SFRS8	4922980	RNA splicing	different		1.78		
SPP1	5403558	synaptic plasticity, etc	different		−2.07		
APBB1	5391818	RNA splicing	different		−1.47		
NDRG4	4329704	cell proliferation, etc	different		−1.54		
PDE7A	5142491	memory	different		2.74		
PIM2	4775974	cell proliferation	different		3.42		

Curation confirmed that 34 DAS genes (40 DAS exons) were reported to be variants or isoforms (see Figure 4A). The probeset ID sequences of 40 DAS exons and previous literature on the variants of these genes were compared to confirm whether the sequence and the variants indicated the same isoforms. The literature was also searched for the distinct functions of the isoforms.

Among the 49 genes with known splicing, 15 genes overlapped with 85 functional genes (Figure 4C). In this study, only the splicing of the Abi2 gene in these 15 genes was analyzed by RT-PCR (Table 2). Although a detailed expression analysis of splicing has not been reported, the RT-PCR and SI results indicated that the skipping isoform (149 nt) of *Abi2* changed to an inclusion isoform (329 nt) during the neuronal differentiation of P19 cells (Table2). Interestingly, one previous study has reported a neural function of *Abi2* gene, showing that *Abi2* is involved in ‘memory’ and ‘learning’ [31]. To test whether *Abi2* splicing is related to this higher brain function, alternative splicing was studied by brain dissection, which revealed that the relative amount of the inclusion isoform was increased in the frontal cortex compared with other tissues (Figure S3). This preliminary result implies that the neural splicing regulation of *Abi2* is quite important for higher brain functions.

## Discussion

One important aspect of Exon Array projects in wet laboratories is that the extracted candidates primarily consist of actual alternative exons validated by RT-PCR. However, there have only been a small number of projects in which the DAS exons were efficiently extracted and validated by RT-PCR [12]. In the present study, 87% of the 262 DAS exons were found to change in the neuronal stage of P19 cells by semiquantitative RT-PCR (Table 2). This score indicates that the filtering conditions used efficiently extracted DAS exons from signals using Exon Arrays. Another important aspect in Exon Array projects is the ease of modifying the filtering conditions. To obtain more reliable DAS exons, the threshold of the ABS_SI_d7_ values in the sixth procedure in Table 1 should be increased (e.g., from ≥1.35 to ≥3.0). A higher ABS_SI value likely indicates a remarkable change of alternative splicing in the RT-PCR experiments (Table 2). Probesets with high ABS_SI_d7_ values indicated alternative exons in a ratio of approximately 40∼50% in the UCSC blat search (Figure 1B). There are probably many alternative splicings in DAS exons with high ABS_SI values. Indeed, these exons can be efficiently validated by RT-PCR in a neural-specific manner.

Although the ABS_SI_d7_ threshold can be variable, we think that a threshold of ABS_SI_d7_ ≥1.35 in the sixth procedure of Table 1 is appropriate. According to the approximation curve, the percentage of predicted alternative exons decreased in the area of high ABS_SI_d7_ values. The percentage of predicted nonalternative exons plateaued at an ABS_SI_d7_ value of approximately 1.35 (Figure 1B). This is because the potential DAS exons accumulated at an ABS_SI_d7_ of ≥1.35. The inflection point between decreasing range and a plateau state (e.g., in in Figure 1B) is probably suitable for use as the ABS_SI threshold. It is also preferable to use accessible software like the Expression Console and Excel. Open access homology search tools such as NCBI BLAST or UCSC blat are useful in the prediction of alternative exons, and reduce the expense of the Exon Array analysis. The extraction method presented here could become the standard method for extracting DAS exons in wet laboratories, because it is useful in projects that employ a small number of samples and promotes the comprehensive analyses of alternative splicing events.

We detected novel splicing patterns in 7 alternative splicing events among 30 randomly selected exons from 262 DAS exons (Figure 2 & Table 2). Therefore, there are still many undetermined alternative splicing events. It is possible that probesets that were removed in the prediction of alternative splicing (Table 1; eighth procedure) also contain novel alternative exons. The latest software or the scoring of exons using exonic splicing enhancer (ESE) and exonic splicing silencer (ESS) may accurately predict DAS exons [32], [33]. Such scoring is especially important for aberrant splicing events, which are frequently observed in cancer cells [34].

In addition to these sequence data, the results of informatics analyses showed that only 49 of the 236 DAS genes have been reported in previous articles of ‘alternative splicing variants’ (Figure 4). Although this informatics analysis is a new trial to identify exon-level annotations, it is likely that insufficient researches has been performed on splicing isoforms. Moreover, there is no assembled database that shows the relationship between a function and each distinct splicing isoform, even though databases for functional analysis are substantial in annotations of genes and proteins. Expression profiles containing alternative splicings are accumulating in databases. Therefore, exon-level informatics analyses will only increase in importance.

Forty-nine of the DAS genes were splicing ‘isoform’ or ‘variant’. Of these, 34 genes had well-studied functions in neural cells or organs. Nine of the 34 genes have a reported distinct function for their alternative isoforms (Table 3). In particular, one Gnao1 isoform, GNAO1 A, is required for light response [30]. *Gnao1* B (189 nt) was a major product in undifferentiated P19 cells and changed to *Gnao1* A (124 nt) in neural differentiated P19 cells (Table 2 & Figure S1). Among adult mouse tissues, *Gnao1* A was abundant in brain or brain dissections (Figure S1 and S3). Therefore, it seems that the splicing regulation of *Gnao1* is primarily involved in neural differentiation. Although *Abi2* is one of the 49 genes with known splicings, it was categorized with the 85 functional genes. Distinct functions for the Abi2 isoforms are unknown. The skipping isoform (149 nt) of *Abi2* was replaced with the inclusion isoform (329 nt) in neuronal differentiated P19 cells (Table2 & Figure S1), and this isoform was largely detected in the frontal cortex of the adult mouse (Figure S1 & S3). Alternative splicing regulation of *Abi2* may be important for higher brain functions, based on the present results and the previous finding that *Abi2* mediates ‘memory’ and ‘learning’ [31].

Along with *Abi2*, the 85 functional genes may be the most important group to investigate alternative splicings, since they are substantially unknown in neural cells or organs. To extract potentially important DAS genes from the 85 functional genes, both alternative splicings and gene expressions were examined. Informatics analyses showed that many DAS genes and DEX-up genes were involved in neural-related events (Figure 3C). Based on the GO biological process terms, we assumed that the DEX-up genes, but not the DEX-down genes, were responsible for events in P19 neuronal cells (Figure 3C). The protein expression levels of the DEX-down genes are probably reduced in P19 neuronal cells. Reductions or disruptions of DEX-down proteins may prevent them from functioning in the differentiated cells.

Some of the DAS genes likely directly or indirectly affected on expressions of the DEX-up genes, and some of the DEX-up genes likely affected on alternative splicings of DAS genes [10]. Therefore, from the pathway analysis, the links between 189 cell processes of DEX-up genes and 85 functional genes out of 236 DAS genes were examined (Figure 4A). Eleven of the 47 presumably important DAS genes mediated the G_1_/S transition in the neuronal differentiation of P19 cells (Figure 5). The regulations of alternative splicing in the 11 DAS genes may play important roles in the cell cycle regulation. This informatics result is very reasonable, since cell proliferation and cell differentiation are highly associated. Some alternative events have been also investigated, but there are still unknown frontiers in alternative splicing research (Table 3).

This study obtained the expression profiles of genes and alternatively spliced transcripts. These profiles included key factors and their potential targets in the transcriptional and alternative splicing networks. Among the DEX-up and DAS genes, we observed 20 and 14 RNA processing-related genes, respectively. These genes may play key roles in neural splicing regulation. For instance, RBM9 was included in both the DAS and DEX-up groups. The splicing activity of the RBM9 isoform, which is indicated by the DAS exon, is reduced compared to that of another isoform [35]. This study also focused on the cell cycle-related genes (Figure 5). SRp38/NSSR1, which we previously detected during neuronal differentiation in P19 cells [22], is a known splicing repressor via dephosphorylation in M-phase specific manner [36]. Nucleophosmin (NPM) is another cell cycle-dependent splicing regulator [37] that is phosphorylated during the G_1_/S transition. These splicing regulators and their potential targets may be very important in future studies clarifying the alternative splicing networks involved in the neuronal differentiation of P19 cells.

## Supporting Information

Figure S1 **Semiquantitative RT-PCR.** (A) Schematic representations of alternative splicing. Thirty of the 262 DAS exons were randomly selected, and 25 alternative splicings are shown above. The remaining splicings are shown in Figure 2. Boxes and middle lines indicate exons and introns, respectively. Gray indicates a possible alternative exon. Arrows indicate locations of the primer annealing sites. Numbers indicate the length of PCR products. The sequences of PCR products were confirmed by sequencing analysis. Semiquantitative RT-PCR during P19 cell differentiation (Day 0, 1, 4, 7, and 10) (B) and in adult mouse brain and other tissues (C). *β-Actin* was used as a control. *GluR1* was used as a neural differentiation marker (B only). Schematic representations of PCR products are shown on the right side of the panels.
(TIF)

Figure S2 **Graphical representations of semiquantitative RT-PCR.** Results of semiquantitative RT-PCR (see Figure 2A, 2B, S1) followed by densitometric analysis are shown in the graphical representation. The amount of each PCR product was divided by total amount in each lane. Percentages are shown in the histogram. Bars indicate the standard error. Numbers on the right side of the graph indicate the length of PCR products. Numbers at the bottom of the graph show the time course: Day 0 (undifferentiated stage), Day 7 (neuronal stage), and Day 10 (early glial stage).
(TIF)

Figure S3 **Alternative splicings in brain dissection.** Semiquantitative RT-PCR experiments were performed using total RNAs of Skeletal muscle, brain, frontal cortex, hippocampus, cerebellum, spinal code (left). Total RNAs of brain dissection are commercially available (Takara). *Gnao1* and *Abi2*, but not *Kif21b*, are categorized among the 49 genes with known splicings. Distinct functions of the *Gnao1* isoforms, but not of the *Abi2* isoforms, were previously reported, but not *Abi2*. *β-Actin* was used as a control. Schematic representations of alternative splicings are shown (right). Boxes and middle lines indicate exons and introns, respectively. Gray indicates a possible alternative exon. Arrows indicate locations of the primer annealing sites. Numbers indicate the length of PCR products.
(TIF)

Table S1 **PCR primers.** Primer sequences, annealing temperatures, and PCR cycles are shown. In the case of ≥3 primers in a DAS exon, multiplex PCR was performed.
(XLS)

Table S2 **List of the 262 DAS exons.**
(XLS)

Table S3 **List of the 1,666 DEX-up genes.**
(XLS)

Table S4 **List of the 550 DEX-down genes.**
(XLS)

Table S5 **List of the 47 DAS genes and their cell processes.**
(XLS)
