# Supplementary figures and images for: Comprehensive Analysis of Alternative Splicing and Functionality in Neuronal Differentiation of P19 Cells
Source: PLoS One. 2011 Feb 18;6(2):e16880. doi: 10.1371/journal.pone.0016880 (PMC3041816; doi:10.1371/journal.pone.0016880)

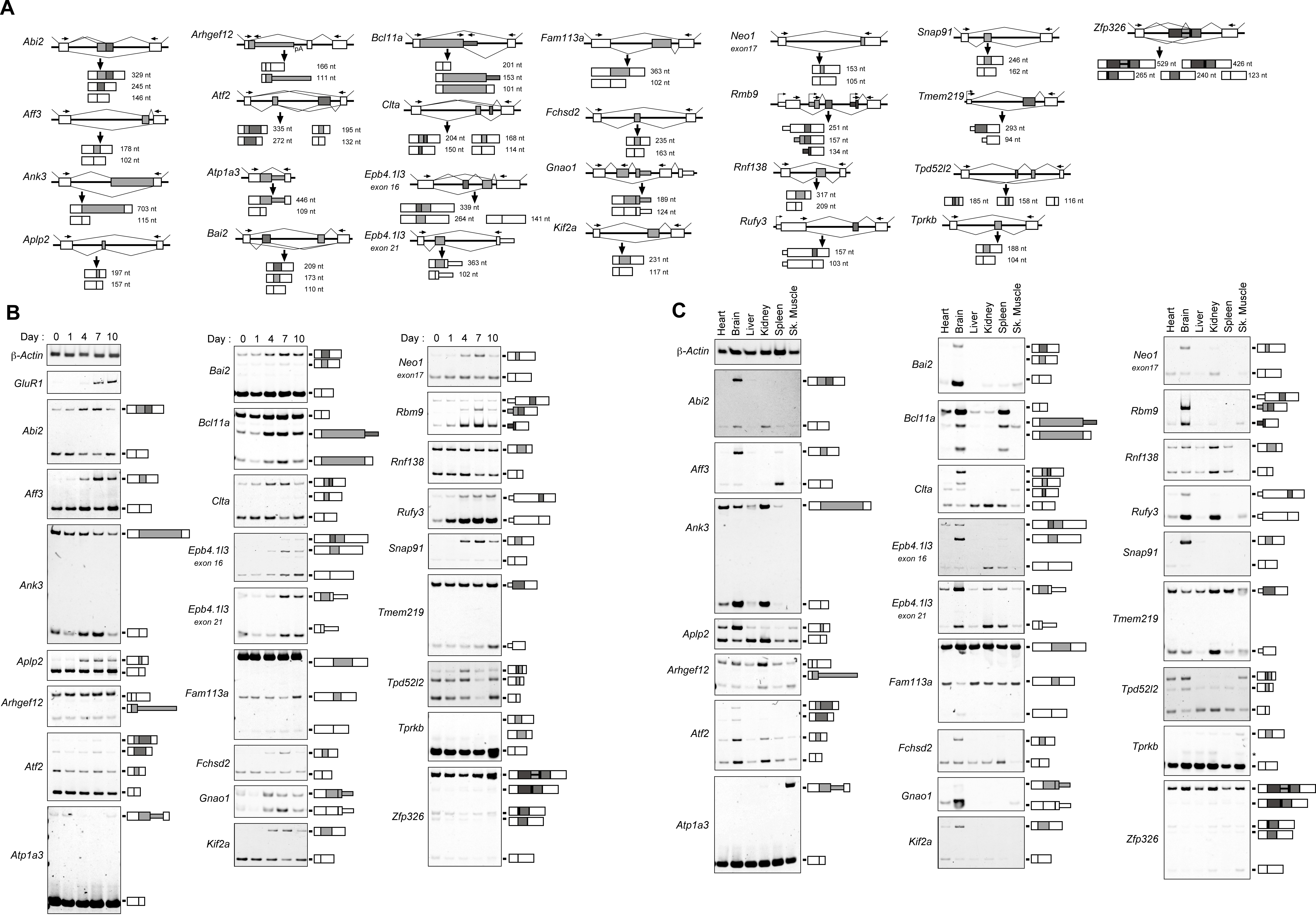

Supplement: Figure S1 — Semiquantitative RT-PCR. (A) Schematic representations of alternative splicing. Thirty of the 262 DAS exons were randomly selected, and 25 alternative splicings are shown above. The remaining splicings are shown in Figure 2. Boxes and middle lines indicate exons and introns, respectively. Gray indicates a possible alternative exon. Arrows indicate locations of the primer annealing sites. Numbers indicate the length of PCR products. The sequences of PCR products were confirmed by sequencing analysis. Semiquantitative RT-PCR during P19 cell differentiation (Day 0, 1, 4, 7, and 10) (B) and in adult mouse brain and other tissues (C). β-Actin was used as a control. GluR1 was used as a neural differentiation marker (B only). Schematic representations of PCR products are shown on the right side of the panels. (TIF) [file pone.0016880.s001.tif]

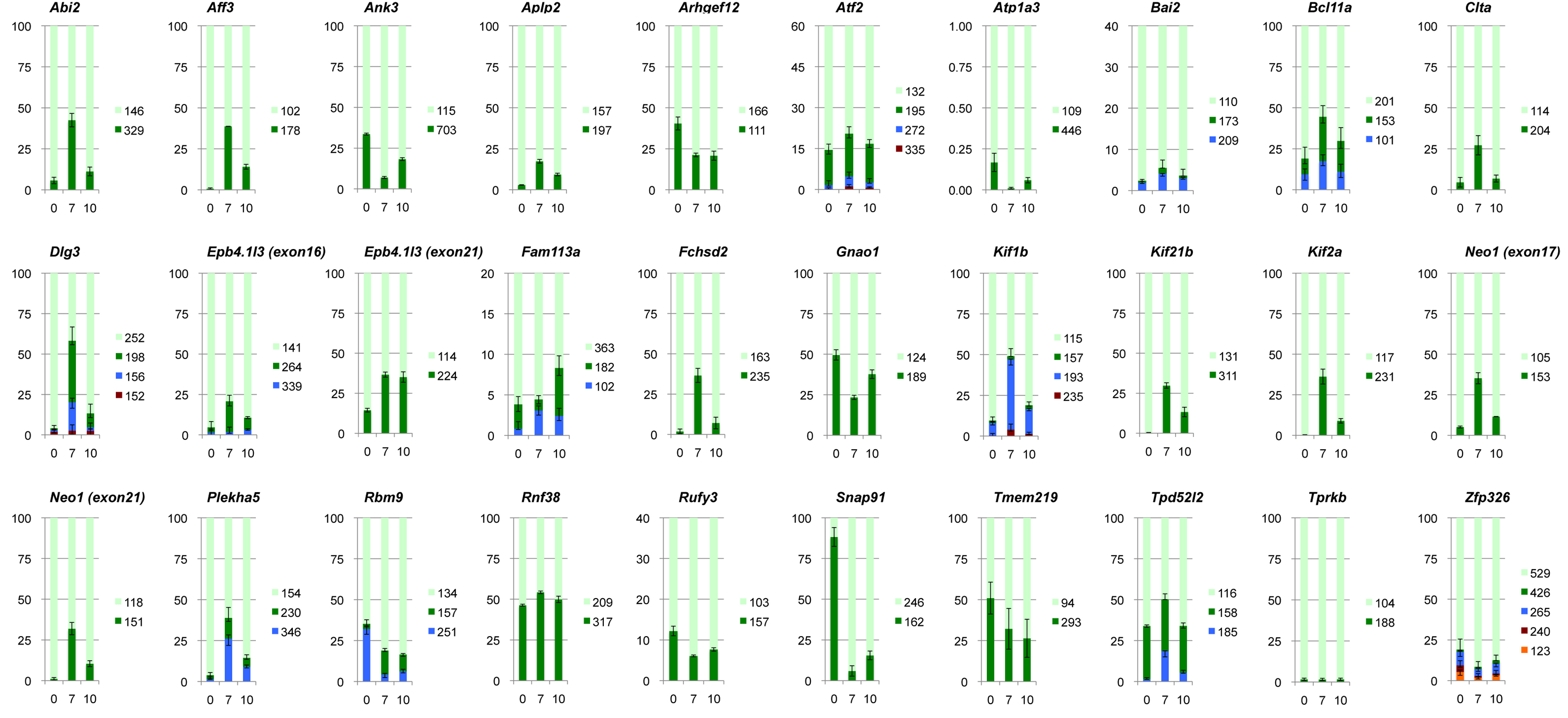

Supplement: Figure S2 — Graphical representations of semiquantitative RT-PCR. Results of semiquantitative RT-PCR (see Figure 2A, 2B, S1) followed by densitometric analysis are shown in the graphical representation. The amount of each PCR product was divided by total amount in each lane. Percentages are shown in the histogram. Bars indicate the standard error. Numbers on the right side of the graph indicate the length of PCR products. Numbers at the bottom of the graph show the time course: Day 0 (undifferentiated stage), Day 7 (neuronal stage), and Day 10 (early glial stage). (TIF) [file pone.0016880.s002.tif]

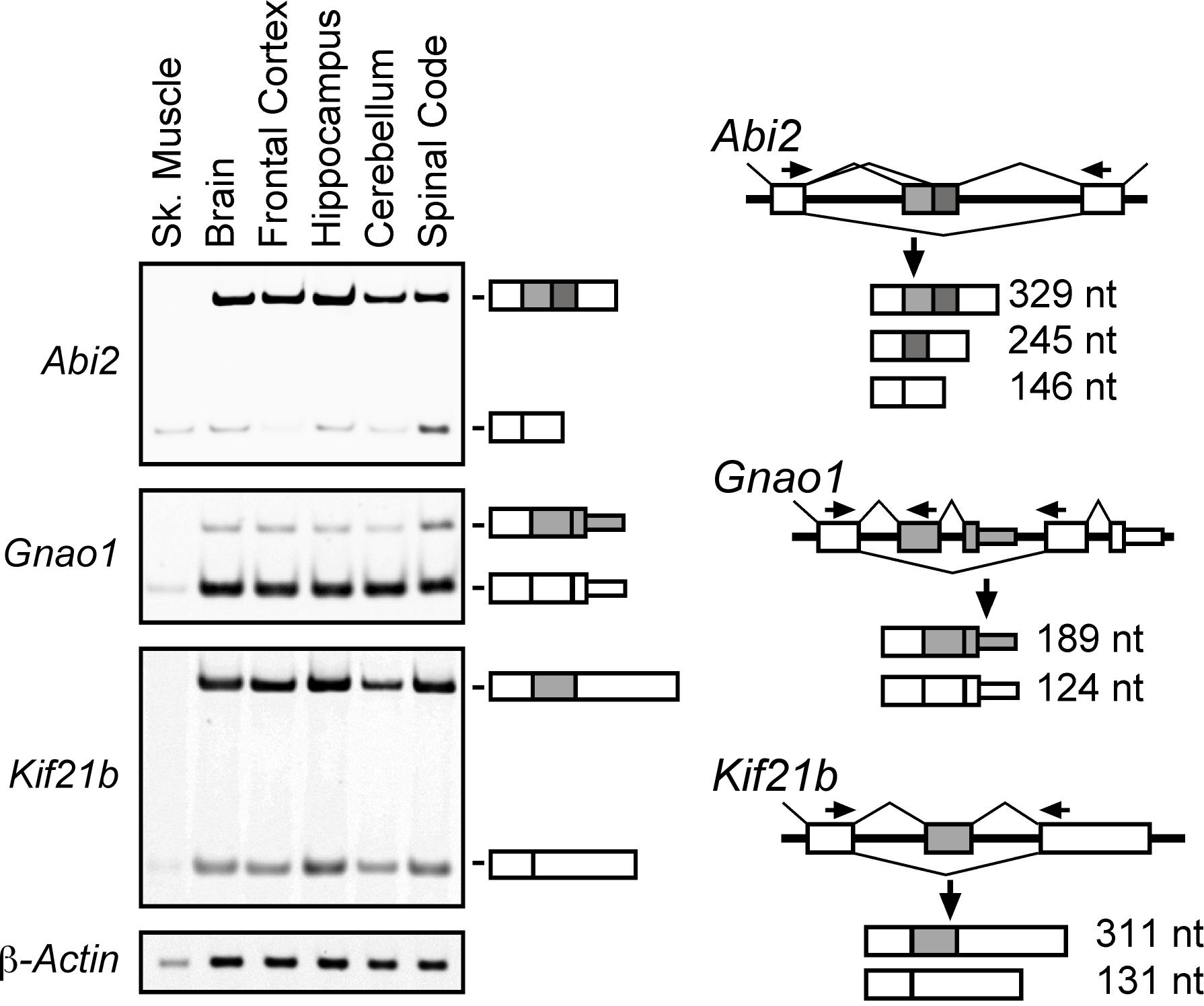

Supplement: Figure S3 — Alternative splicings in brain dissection. Semiquantitative RT-PCR experiments were performed using total RNAs of Skeletal muscle, brain, frontal cortex, hippocampus, cerebellum, spinal code (left). Total RNAs of brain dissection are commercially available (Takara). Gnao1 and Abi2, but not Kif21b, are categorized among the 49 genes with known splicings. Distinct functions of the Gnao1 isoforms, but not of the Abi2 isoforms, were previously reported, but not Abi2. β-Actin was used as a control. Schematic representations of alternative splicings are shown (right). Boxes and middle lines indicate exons and introns, respectively. Gray indicates a possible alternative exon. Arrows indicate locations of the primer annealing sites. Numbers indicate the length of PCR products. (TIF) [file pone.0016880.s003.tif]
